# Scleral onlay pericardial graft for traumatic globe rupture: A case report

**DOI:** 10.1097/MD.0000000000047886

**Published:** 2026-03-13

**Authors:** Junkyu Chung, Jin-Ho Joo

**Affiliations:** a Department of Ophthalmology, Kyung Hee University Hospital at Gangdong, Kyung Hee University, Seoul, South Korea; b Department of Ophthalmology, Chung-Ang University College of Medicine, Seoul, South Korea; c Department of Ophthalmology, Chung-Ang University Gwangmyeong Hospital, Gwangmyeong, Gyeonggi-do, South Korea.

**Keywords:** globe rupture, ocular trauma, open globe injury, pericardial graft

## Abstract

**Rationale:**

Primary watertight scleral closure is the standard approach for the treatment of open globe injuries. However, in cases with posterior globe rupture, surgical access may be severely limited, making direct suturing impossible. In such cases, alternative methods are required to preserve globe integrity and prevent phthisis. This report describes a rare case of posterior globe rupture, for which primary scleral suturing was not feasible, successfully managed with a scleral onlay human pericardial graft.

**Patient concerns:**

A 75-year-old woman presented with no light perception vision and total hyphema after blunt trauma to the right eye.

**Diagnoses:**

Imaging suggested a zone III globe rupture located posterior to the equator with inverted scleral margins, which was confirmed by exploratory surgery.

**Interventions:**

Emergency surgery was performed under general anesthesia. A preserved human pericardial graft was trimmed and placed in an onlay fashion to cover the scleral wound. Only the anterior graft margin was sutured to the sclera, and a 2nd pericardial graft was overlaid to provide additional tectonic support. The conjunctiva was repositioned over the graft.

**Outcomes:**

Postoperatively, intraocular pressure and globe contour were maintained for at least 4 months, although vision did not improve. No signs of infection or graft displacement were observed during the follow-up.

**Lessons:**

A scleral onlay pericardial graft with minimal anterior fixation can be a valuable technique for preserving ocular integrity in posterior globe ruptures in which primary closure is not possible. This approach may help to prevent phthisis and maintain the ocular surface for future prosthetic rehabilitation.

## 1. Introduction

Standard management of open globe injuries is to achieve a watertight closure.^[1]^ However, when a rupture involves the posterior sclera, surgical access can be severely limited. Wounds located behind the equator may require substantial manipulation of the globe for visualization, which can be technically challenging and potentially harmful. In such situations, suturing may only be performed on the accessible anterior portion of the wound, whereas the posterior extent is left untreated. Alternatively, the defect can be covered with a pericardial or scleral patch graft to provide tectonic support and maintain globe integrity.^[1]^

Various materials – including preserved pericardium, donor sclera, and synthetic patches – have been used as ocular reinforcement grafts.^[2,3]^ Donor sclera offers strong tectonic support but may show variable thickness, long-term thinning or resorption, and a potential risk of viral transmission.^[3]^ Synthetic materials such as expanded polytetrafluoroethylene provide sterility, uniform thickness, and favorable tissue integration due to their porous structure, although thicker sheets may induce conjunctival tension and melting.^[2]^ In contrast, preserved pericardium is thin, flexible, immune-tolerant, and well-integrated with surrounding tissues.^[3]^

Previous reports have described the use of preserved pericardial grafts for globe rupture in specific cases, such as reinforcing the thinned sclera, closing ruptured trabeculectomy sites, and repairing scleral ruptures during retinal detachment.^[4–7]^ However, no prior reports have described the management of posterior globe rupture by covering the inaccessible area with an onlay pericardial graft, suturing only its anterior margin, and repositioning the conjunctiva over it. The use of this minimal-fixation onlay technique with a pericardial graft preserved globe integrity in the present case.

Written informed consent was obtained from the patient for the publication of this case report. Ethical committee approval was not required as this was a retrospective description of a single case and did not involve prospective research procedures.

## 2. Case report

**Figure 1.**
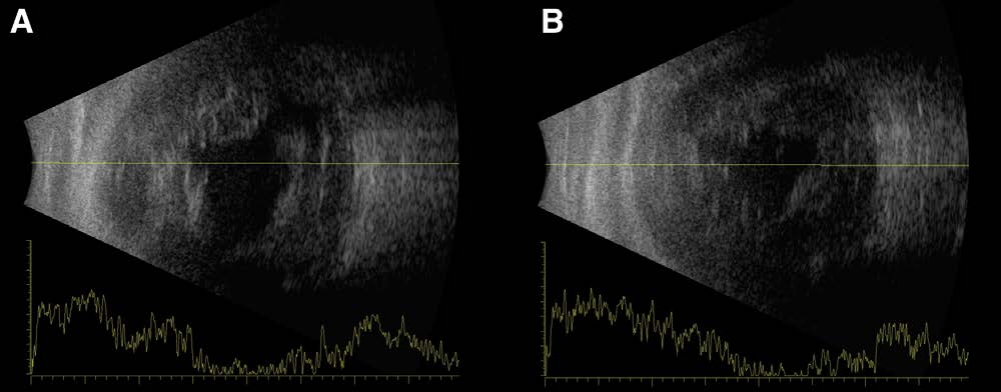
Preoperative B-scan ultrasonography of the right eye. (A) Horizontal and (B) vertical scans showing choroidal detachment and vitreous hemorrhage.

**Figure 2.**
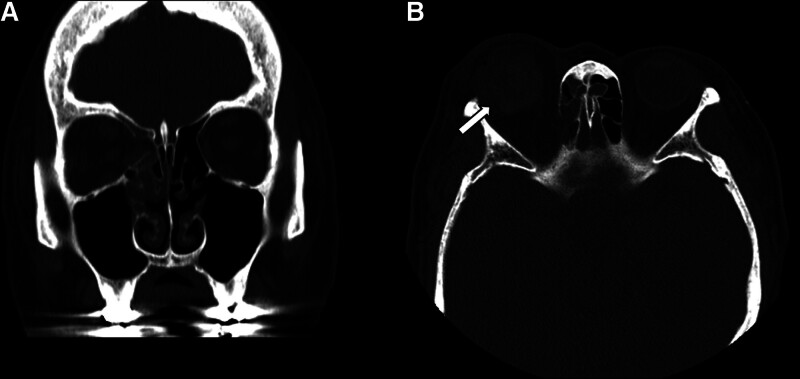
Preoperative computed tomography of the orbit. (A) Coronal view showing a medial orbital wall fracture. (B) Axial view demonstrating the site of posterior temporal scleral rupture with suspected rolled margin (arrow).

A 75-year-old female patient with a history of variant angina, but no prior ophthalmic history sustained blunt trauma to her right eye after falling and striking a bicycle pedal. On presentation, the intraocular pressure (IOP) was 24 mm Hg, measured by Goldmann applanation tonometry and the visual acuity was no light perception. Slit-lamp examination showed apparently intact conjunctiva but total hyphema, which precluded a posterior segment view. B-scan ultrasonography revealed choroidal detachment and vitreous hemorrhage (Fig. 1). Computed tomography showed a medial orbital wall fracture and suspected posterior temporal scleral rupture with inwardly rolled margins (Fig. 2).

**Figure 3.**
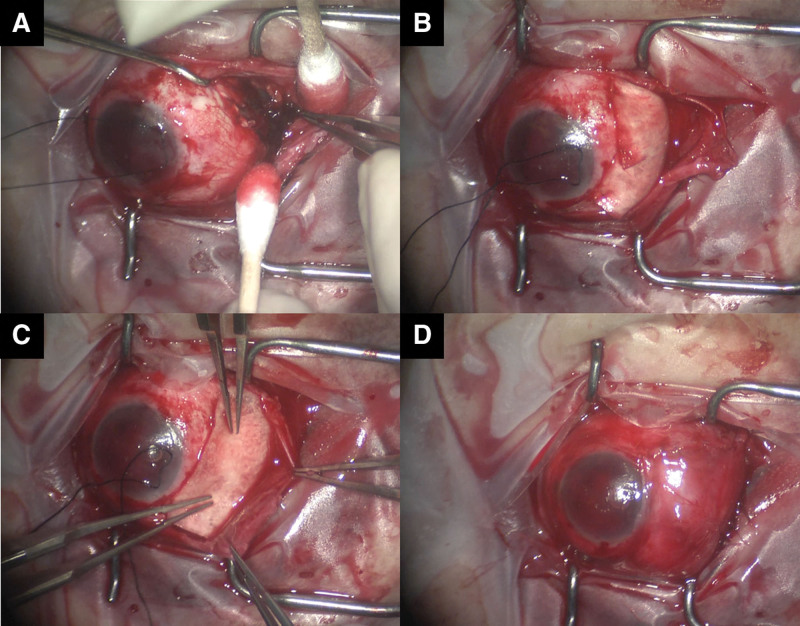
Intraoperative photographs of posterior globe rupture repair using an onlay pericardial graft. (A) Circumferential scleral rupture posterior to the equator with massive vitreous and uveal tissue loss. (B) Placement of the 1st hydrated pericardium graft in an onlay fashion, secured only at the anterior margin. (C) Placement of the 2nd pericardium graft over the 1st graft for additional tectonic support. (D) Surgery completion after conjunctival closure over both grafts.

Emergency exploration was performed under general anesthesia. A localized conjunctival peritomy was made to expose the rupture site as far posteriorly as possible, minimizing manipulation to prevent further intraocular prolapse. The rupture was circumferential and located posterior to the equator, with massive vitreous and uveal tissue loss (Fig. 3A). The posterior scleral margin was inverted and inaccessible, making direct scleral suturing impossible without risking further tissue loss.

A preserved lyophilized human pericardium graft (Solvita, Dayton) was hydrated and trimmed to overlap the healthy sclera beyond all visible margins of the rupture. The 1st graft was placed in an onlay fashion over the wound and secured only at its anterior margin to the sclera with interrupted 8-0 Vicryl sutures (Ethicon, Inc., Somerville) to prevent anterior displacement. The posterior portion was left unfixed to avoid deep manipulation in the zone of maximal tissue loss (Fig. 3B). A 2nd graft of similar size was then placed directly over the 1st graft without additional scleral fixation, serving as an extra protective layer (Fig. 3C). The conjunctiva was repositioned over both grafts and closed using interrupted 8-0 Vicryl sutures (Fig. 3D).

**Figure 4.**
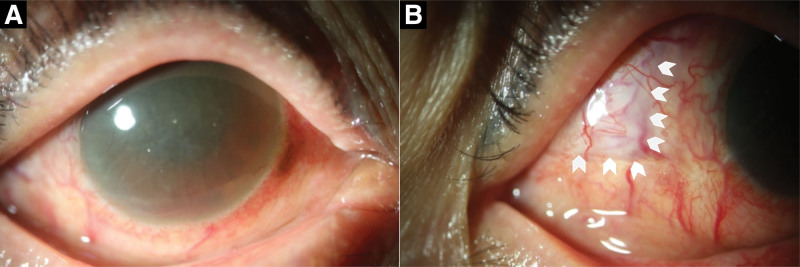
Anterior segment photographs at 4 months postoperatively. (A) Frontal view showing a preserved globe contour and quiet anterior chamber. (B) Temporal conjunctiva in the closed position covering the pericardial graft (arrowheads indicate the margins of the underlying graft).

On postoperative day 1, the anterior chamber was deep, the IOP remained at 24 mm Hg, and the vision remained no light perception. At 2 months, the IOP was 16 mm Hg, the anterior chamber was stable, and the globe contour was preserved, although the visual function did not recover. Four months after surgery, the IOP was stable at 15 mm Hg, the anterior chamber maintained its depth, and globe contour remained stable (Fig. 4). No signs of infection or graft displacement were noted during the follow-up. The timeline of the patient injury, diagnosis, intervention, and outcomes is summarized in Table 1. To prevent postoperative wound infection, systemic antibiotics were administered for 1 week and topical antibiotics were prescribed for 1 month, along with topical corticosteroids to control inflammation and support graft stability. The patient adhered well to the postoperative regimen and follow-up schedule, and tolerated the treatment without major complications. From the patient perspective, retaining the globe provided significant psychological comfort. She stated, “Although I cannot see, I am grateful that I did not lose my eye and still look like myself.,” expressing high satisfaction with the cosmetic outcomes despite the permanent visual loss.

**Table 1:** Timeline of the case.

Timeline	Events and interventions	Outcomes
Injury	Blunt trauma to the right eye caused by striking a bicycle pedal.	Pain and vision loss.
Initial presentation	Emergency department visit immediately after injury.	VA: NLP. IOP: 24 mm Hg. Slit-lamp: Total hyphema. B-scan: Choroidal detachment, vitreous hemorrhage. CT: Medial orbital wall fracture, posterior scleral rupture.
Intervention (day 0)	Emergency surgical exploration under general anesthesia. Repair using double-layered onlay pericardial grafts with anterior marginal fixation.	Successful coverage of the posterior scleral defect.
Postoperative day 1	Bedside examination.	VA: NLP. IOP: 24 mm Hg. Anterior chamber: Deep formed.
Postoperative month 2	Follow-up examination.	VA: NLP. IOP: 16 mm Hg. Anterior chamber: Stable. Globe contour: Preserved.
Postoperative month 4	Follow-up examination.	VA: NLP. IOP: 15 mm Hg. Globe contour: Stable. No signs of infection, graft displacement, or phthisis.

CT = computed tomography, IOP = intraocular pressure, NLP = no light perception, VA = visual acuity.

## 3. Discussion

In ophthalmic surgery, preserved pericardium is frequently selected as a patch graft because it is durable, well tolerated, and immunogenically inert.^[8,9]^ This method is most often applied in glaucoma drainage device surgery to cover the tube, and it has also been used in various situations to reinforce the sclera after strabismus surgery, repair ruptured trabeculectomy sites following trauma, repair scleral rupture during retinal detachment surgery, close corneal perforations, and prevent orbital implant exposure.^[4–7,9–12]^ These reports demonstrate that the pericardium is a readily available, sterile material that can provide durable tectonic support. Beyond availability, pericardial grafts are particularly advantageous over donor sclera in scenarios involving irregular, friable, or necrotic wound edges, such as ruptured trabeculectomy blebs or severe scleral thinning following strabismus surgery.^[5,6]^ Unlike donor sclera, which is thicker (~1 mm) and more rigid, processed pericardium has a thinner profile (~400 µm) and possesses a multidirectional matrix. These properties facilitate easier handling and secure suturing without the risk of “cheese-wiring” through delicate tissues.^[5]^ Regarding long-term durability, indirect evidence from glaucoma tube shunt coverage and orbital implant repair suggests that while pericardial grafts undergo thinning over time – up to 26% thinning observed at a mean follow-up of 32.6 months – they maintain effective structural integrity with low rates of erosion or exposure over follow-up periods ranging from 1 to 3 years.^[10,11]^

The scleral rupture in the present case was located posterior to the equator, with an inverted and inaccessible posterior margin, making standard watertight primary closure impossible. As extensive posterior dissection to expose the wound was potentially harmful and technically unfeasible, a preserved human pericardial graft was instead placed in an onlay fashion over the rupture and secured only along the anterior margin. This minimal fixation avoided deep posterior manipulation while covering the entire defect. Because additional manipulation of the posterior sclera increased the risk of further vitreous and uveal tissue loss, we elected not to place sutures posteriorly and avoided excessive handling of the area. A 2nd onlay graft was added for additional tectonic support, and the conjunctiva was repositioned over the graft. The sclera in humans is measured to be between 0.39 and 1.0 mm thick, yet a portion of the rupture site was expected to be covered solely by the pericardial graft without underlying native sclera.^[13]^ Considering that pericardial grafts are reported to thin postimplantation, reaching as low as 0.115 mm in certain areas after 1 year, a double-layered application was deemed necessary to provide sufficient tectonic support and compensate for this expected long-term thinning.^[14]^

This technique provided immediate structural reinforcement in a situation where posterior scleral closure was not possible, maintaining the globe contour and intraocular pressure for at least 4 months postoperatively, although vision could not be restored. The main advantages of this technique are its potential to prevent phthisis and preserve the ocular surface for future prosthetic rehabilitation. The limitations include the inability to restore vision in cases with severe intraocular tissue loss, and the relatively short follow-up period. Although the graft remained stable at 4 months, late-onset complications cannot be definitively excluded. Potential long-term risks include graft resorption, conjunctival dehiscence, delayed infection, or progressive uveal atrophy. Further studies are needed to assess the long-term outcomes and compare this method with other approaches for posterior globe rupture.

The use of a pericardial graft was justified by its material properties, which minimize the risks inherent in utilizing ocular patch grafts, such as thinning, melting, and the devastating complication of infection. Pericardial grafts, being chemically treated and gamma-irradiated, offer a guarantee of sterility, reducing the initial risk of pathogen transmission.^[10]^ Clinically, these grafts are reported to show good biocompatibility and are less susceptible to melting compared to donor sclera in other ocular surgeries.^[3,10]^ In the present case, our 4-month follow-up confirmed the material integrity, showing no signs of infection or graft displacement.

Ultimately, the technique greatest value lies in its ability to avoid enucleation by successfully maintaining the globe contour. The absence of complications and the preserved globe form observed up to 4 months postoperatively demonstrate the successful achievement of the primary surgical goal: ensuring ocular integrity and maintaining the ocular surface for future prosthetic rehabilitation.

Importantly, the patient reported satisfaction with the preserved globe despite the lack of visual recovery, indicating that ocular integrity itself can provide meaningful psychological and social benefit by avoiding enucleation and supporting quality of life. Enucleation-related anophthalmia can negatively affect patients in several ways, including reduced quality of life related to visual functioning and heightened concerns about cosmetic changes. Individuals with this condition also tend to report anxiety and depressive symptoms more frequently than the general population.^[15]^ This perspective highlights an additional value of the technique beyond structural reinforcement, as maintaining the globe contour avoids the negative psychosocial impact of eye loss and provides a foundation for optimal future prosthetic rehabilitation and psychological comfort.

## 4. Conclusion

When posterior globe ruptures cannot be closed using direct scleral suturing, an onlay pericardial graft secured only at the anterior margin offers a practical and safe alternative. By minimizing posterior manipulation, this technique reduces surgical risk while providing sufficient tectonic support to preserve globe contour and IOP.

## Acknowledgments

We would like to thank Editage (www.editage.co.kr) for the English language editing.

## Author contributions

**Conceptualization:** Junkyu Chung, Jin-Ho Joo.

**Data curation:** Junkyu Chung.

**Formal analysis:** Junkyu Chung.

**Investigation:** Junkyu Chung.

**Methodology:** Junkyu Chung, Jin-Ho Joo.

**Project administration:** Junkyu Chung, Jin-Ho Joo.

**Writing – original draft:** Junkyu Chung.

**Writing – review & editing:** Jin-Ho Joo.
